# Double Product and Autonomic Function as Predictors of Quality of Life in Heart Transplant Recipients: A Cross-Sectional Study

**DOI:** 10.21470/1678-9741-2021-0083

**Published:** 2022

**Authors:** Luiz Fernando Rodrigues Junior, Beatriz Robert Moreira, Alice Pereira Duque, Juliana Rega de Oliveira, Pedro Henrique Scheidt Figueiredo, Cláudia Rosa de Oliveira, Alexandre Siciliano Colafranceschi, Mauro Felippe Felix Mediano, Tereza Cristina Felippe Guimarães

**Affiliations:** 1 Physiotherapy Service, Instituto Nacional de Cardiologia, Rio de Janeiro, Rio de Janeiro, Brazil; 2 Department of Physiological Sciences, Universidade Federal do Estado do Rio de Janeiro, Rio de Janeiro, Rio de Janeiro, Brazil; 3 Education and Research Department, Instituto Nacional de Cardiologia, Rio de Janeiro, Rio de Janeiro, Brazil; 4 Postgraduate Program in Rehabilitation and Functional Performance, Universidade Federal dos Vales do Jequitinhonha e Mucuri, Diamantina, Minas Gerais, Brazil; 5 Laboratory of Clinical Research on Chagas Disease, Instituto Nacional de Infectologia Evandro Chagas, Fundação Oswaldo Cruz, Rio de Janeiro, Rio de Janeiro, Brazil

**Keywords:** Heart Transplantation, Quality Of Life, Autonomic Nervous System, Heart Rate Variability, Hemodynamics.

## Abstract

**Introduction:**

Heart rate control by the autonomic nervous system (ANS) is impaired in heart transplant (HT) recipients, leading to increased resting heart rate, metabolic demand, and fatigue, which can impair their quality of life (QoL). In this study, we hypothesized the association of hemodynamics and autonomic function as predictors of QoL in HT recipients.

**Methods:**

This is a cross-sectional study conducted with HT recipients aged ≥ 18 years at ambulatorial accompaniment. Blood pressure was used for hemodynamics assessment, and heart rate variability (HRV) was used for ANS assessment. QoL was assessed by the 36-item Short Form Health Survey. The significance level was set as P≤0.05.

**Results:**

Twenty-two volunteers were included in the study. Systolic blood pressure (SBP) and double product (DP) were significantly negatively associated with the physical functioning domain of QoL. DP, the number of consecutive normal RR interval differences > 50 ms (NN50), and the percentage of normal RR intervals that differed by > 50 ms from the adjacent interval (PNN50) exhibited negative association with the physical role domain. NN50 and PNN50 were significantly associated with bodily pain, social functioning, and emotional role domains. SBP was negatively associated with the vitality domain. Considering general and mental health domains, no variable demonstrated significant association. DP, NN50, and PNN50 were negatively associated with the total score of QoL.

**Conclusion:**

This study demonstrated DP and HRV as predictors of QoL in HT recipients. These innovative results can become a relevant therapeutic target for improving QoL in HT recipients prior to its deterioration.

**Table:** 

Abbreviations, Acronyms & Symbols			
ANS BP	= Autonomic nervous system = Blood pressure	PNN50	= Percentage of normal RR intervals that differed by > 50 ms from the adjacent interval
CI	= Confidence interval	QoL	= Quality of life
DBP	= Diastolic blood pressure	RMSSD	= Root-mean-square of successive differences
DP	= Double product	SANS	= Sympathetic autonomic nervous system
HF	= High frequency component	SBP	= Systolic blood pressure
HR	= Heart rate	SD	= Standard deviation
HRV HT	= Heart rate variability = Heart transplant	SD1	= Standard deviation of instantaneous beat-to-beat interval variability
LF	= Low frequency component	SD2	= Continuous long-term RR interval variability

## INTRODUCTION

Heart failure - the final stage of most heart diseases and the main cause of death of patients with ischemic heart disease in the United States of America and other Western countries^[1]^ - is characterized by the inability of the heart to provide adequate blood supply to all tissues of the body, impairing patients’ functional capacity and quality of life (QoL). As the disease advances further, morbidity and mortality increase^[2^,^3]^. Therapeutic refractory heart failure necessarily culminates in death, being heart transplant (HT) the only alternative to avoid this outcome^[4]^. This treatment has immeasurable benefits such as prolonging survival by approximately 10 years following surgery and also improving symptoms of heart failure, enabling a more active lifestyle and improving QoL for the 85% of individuals that survive the first year after HT surgery^[5]^.

However, during surgical procedure, transplanted hearts are implanted without afferent and efferent nerve connections, remaining completely denervated for approximately one year. Consequently, heart rate (HR) control by the autonomic nervous system (ANS) is impaired, leading to increased resting HR, increased metabolic demand, and fatigue, which can negatively impact QoL of HT recipients due physiological, clinical, and behavioral profile impairments, such as the need for more daily-use medications for HR controlling^[6^,^7]^. Usually, there are evidence of reinnervation occurring in the second year after surgery, reaching myocardial muscle, sinoatrial node, and coronary vessels, but remaining incomplete and regionally limited many years after the transplant. Nevertheless, restoration of cardiac innervation can improve exercise capacity as well as blood flow regulation in the coronary arteries, and hence improve QoL^[8]^.

In the present study, we hypothesized the association of hemodynamics and autonomic function as predictors of QoL in HT recipients, which could be future monitoring and therapeutic target for improving QoL of this population.

## METHODS

### Study Design

This is a cross-sectional study conducted from January to July 2017 using baseline data from a previously published clinical trial^[9]^. All patients in ambulatorial accompaniment after heart transplantation at the Instituto Nacional de Cardiologia were eligible for the study. After phone contact, those ≥ 18 years old who accepted to participate were included, and those not able to presently respond the questionnaires were excluded from the study.

### Ethical Considerations

This study was conducted in accordance with the Helsinki Declaration of 1975, as revised in 2013. The Ethics Committee of the Instituto Nacional de Cardiologia (CAAE: 61053316.0.0000.5272) approved the study, and all patients signed an informed consent form before the beginning of the data collection.

### Measurements

All the information was collected in person, after ambulatorial medical appointment, on individual assessment sheets filled out by the same researcher previously trained to perform all the study procedures. The following data were collected: age, gender, history of previous and current disease, comorbidities, and QoL. Assessments of HR, blood pressure (BP), and heart rate variability (HRV) were also performed.

### QoL Assessment

The Portuguese validated version of 36-item Short Form Health Survey (SF-36) was used to assess health-related QoL^[10]^. The SF-36 is divided in eight dimensions: physical functioning, physical role, bodily pain, general health, vitality, social functioning, emotional role, and mental health. Also, the summary Physical Composite Score (comprising physical functioning, physical role, bodily pain, and general health) and Mental Composite Score (comprising vitality, social functioning, emotional role, and mental health) were calculated and used to obtain the total QoL score, representing the mean value of the Physical and Mental Composite Scores^[10^,^11]^. The scores ranged from 0 to 100, with higher values denoting better functioning and well-being.

### Heart Rate, Blood Pressure, and Heart Rate Variability Measurements

The resting HR, BP, and HRV measurements were carried out in a quiet room with a controlled temperature (23°C). HR, systolic blood pressure (SBP), diastolic blood pressure (DBP), and mean arterial pressure (MAP) were registered after a 20-minute resting period, in the sequence of the interview, and anthropometric measurements were obtained with the patients at supine position. BP, HR, and RR interval were registered. The double product (DP) was calculated as a product of HR and SBP.

The RR intervals were continuously recorded throughout a 10-minute period after resting using a HR monitor (RS800; Polar, United States of America) and subsequently used to evaluate HRV using appropriate software (Kubios v.2.2; University of Eastern Finland, Finland). The sympathetic autonomic nervous system (SANS) and parasympathetic autonomic nervous system (PANS) activity were quantified using HRV by calculating the following time-domain parameters: mean of all normal RR interval (mean RR, associated to SANS and PANS modulations), standard deviation of normal RR interval (or SDNN, associated to activity of both SANS and PANS), number of consecutive normal RR interval differences > 50 ms (NN50, associated to the PANS activity), percentage of normal RR intervals that differed by > 50 ms from the adjacent interval (PNN50, the proportion of NN50 divided by total number of NN, which also represents the PANS activity), root-mean-square of successive differences (or RMSSD, associated to the PANS activity), triangular index (total number of all NN intervals divided by the height of the histogram of all NN intervals measured on a discrete scale with bins of 7.8125 ms [1/128 seconds]), and the baseline width of the minimum square difference triangular interpolation of the highest peak of the histogram of all NN intervals (or TINN). Also, the integration of the successive HR bands was classified in relation to the frequency domain as follows: very low frequency component (0.003-0.04 Hz; related to renin-angiotensin-aldosterone system, thermoregulation, peripheral vasomotor tonus, and PANS activity), low frequency component (LF; 0.04-0.15 Hz; representing the SANS and PANS activity, with a predominance of SANS influence), and high frequency component (HF; 0.15-0.4 Hz; associated with the PANS activity). The ratio of LF to HF (LF/HF) was used to calculate the sympathovagal index. The normalized power of the LF and HF components (representing total HRV) was calculated in standard units (nu). Finally, nonlinear analysis of HRV was made using the Poincaré plot to calculate the standard deviation of instantaneous beat-to-beat interval variability (SD1, associated to the PANS activity), the continuous long-term RR interval variability (SD2, representing total HRV), and the SD1/SD2 ratio (SD12)^[12^,^13]^.

### Data Analysis

Descriptive analysis consisted of mean and standard deviation (SD) for continuous variables and percentage and number of observations for categorical variables. The association between the variables was determined using linear regression models with each QoL domain (functional capacity, physical aspects, pain, general health status, vitality, social aspects, emotional aspects, mental health, and total score) as the dependent variable, and the physiological measurements (HR, SBP, DBP, MAP, DP, HRV variables) as independent variables. Models were fitted without adjustments and adjusted for age, gender, and time since surgery, considering the role of these variables as potential confounders. The *P*-value < 0.05 was considered statistically significant.

## RESULTS

A total of 22 volunteers were included in the study, five women (22.7%) and 17 men (77.3%), with mean age of 53.4 (9.3) years old. The HT indications were: chagasic cardiomyopathy, myocarditis, ischemic heart disease, and valvar dysfunction due to rheumatic disease, each with four cases (18.2%); and alcoholic, idiopathic, and peripartum cardiomyopathy with three (3.7%), two (9.1%), and one case (4.5%), respectively. Also, the mean time since surgery was 4.3 (2.5) years. The main comorbidities were hypertension (50.0%), diabetes (31.8%), obesity (27.2%), dyslipidemia (22.7%), hypothyroidism (13.7%), chronic kidney disease (13.7%), and hyperthyroidism (4.5%).

Table 1 shows the crude means and SD of hemodynamics, the time, frequency, and nonlinear domains of HRV, and QoL domains of the studied sample. The non-adjusted association analysis is presented in Tables S1 and S2.

**Table 1:** Baseline characteristics of patients included in the study.

Variable	Mean ± SD or Number (%)
**Hemodynamics**	
HR (bpm)	78.8±6.9
SBP (mmHg)	124.6±11.9
DBP (mmHg)	84.0 (7.3)
MAP (mmHg)	101.0 (10.0)
DP (bpm.mmHg)	9802.9±1104.2
**HRV**	
*Time domain*	
Mean RR (ms)	769.6±71.6
SDNN (ms)	7.2±3.0
RMSSD (ms)	6.1±3.4
NN50 (number)	0.1±0.3
PNN50 (%)	0.2±0.1
Triangular index	2.5±0.7
TINN (ms)	28.6±19.6
*Frequency domain*	
Total power (ms^^2^^)	
VLF (ms^^2^^)	34.4±45.3
LF (nu)	37.6±22.5
HF (nu)	61.4±21.4
LF/HF	0.9±0.7
*Nonlinear*	
SD1	3.8±2.3
SD2	9.4±3.9
SD1/SD2 ratio	0.4±0.2
**Quality of life (SF-36)**	
Physical functioning	77.9±21.7
Physical role	78.4±41.0
Bodily pain	72.8±30.2
General health	64.6±15.4
Vitality	74.1±23.4
Social functioning	86.4±16.8
Emotional role	93.9±16.7
Mental health	77.8±16.7
Physical health composite	73.44±21.6
Mental health composite	83.0±14.2
Total score	78.2±17.5

DBP=diastolic blood pressure; DP=double product; HF=high frequency component; HR=heart rate; HRV=heart rate variability; LF=low frequency component; LF/HF=low frequency/high frequency ratio (sympathovagal index); MAP=mean arterial pressure; NN50=number of consecutive normal RR interval differences > 50 ms; PNN50=percentage of normal RR intervals that differed by > 50 ms from the adjacent interval; RMSSD=root-mean-square of successive differences; SBP=systolic blood pressure; SD=standard deviation; SD1=standard deviation of instantaneous beat-to-beat interval variability; SD2=continuous long-term RR interval variability; SDNN=standard deviation of normal RR interval; SF-36=36-item Short Form Health Survey; TINN=baseline width of the minimum square difference triangular interpolation of the highest peak of the histogram of all NN intervals; VLF=very low frequency component

**Table S1:** Associations between hemodynamics and heart rate variability variables with SF-36 Physical Composite domains (non-adjusted analysis).

	Physical functioning			Physical role			Bodily pain			General health		
	β	95% CI	*P*-value	β	95% CI	*P*-value	β	95% CI	*P*-value	β	95% CI	*P*-value
**Hemodynamics**												
HR (bpm)	-0.4	-1.5 to +0.6	0.404	-1.6	-3.6 to +0.3	0.101	-0.6	-2.1 to +0.9	0.397	+0.1	-0.7 to +0.8	0.898
**SBP (mmHg)**	**-0.7**	**-1.3 to -0.1**	**0.019**	-0.8	-2.1 to +0.4	0.177	-0.8	-1.6 to 0.1	0.096	-0.1	-0.6 to +0.4	0.725
DBP (mmHg)	-0.2	-1.3 to + 0.9	0.728	-1.0	-3.0 to +1.0	0.321	-0.9	-2.4 to +0.5	0.200	+0.1	-0.7 to +0.8	0.957
MAP (mmHg)	-0.6	-1.6 to +0.3	0.195	-1.1	-3.0 to +0.7	0.208	-1.0	-2.4 to +0.3	0.112	-0.1	-0.7 to +0.7	0.891
**DP (bpm.mmHg)**	**-0.1**	**-0.1 to 0.0**	**0.024**	**-0.1**	**-0.1 to 0.0**	**0.008**	-0.1	-0.1 to +0.1	0.168	-0.1	-0.1 to +0.1	0.557
**HRV**												
**Time domain**												
Mean RR (ms)	-0.1	-.17 to .11	0.697	+0.1	-02 to +0.4	0.443	-0.1	-0.2 to +0.1	0.484	-0.1	-0.1 to +0.1	0.751
SDNN (ms)	+ 2.3	-0.8 to +5.5	0.148	+2.9	-3.3 to +9.1	0.337	-0.1	-4.8 to +4.6	0.961	+0.4	-2.0 to +2.7	0.749
RMSSD (ms)	+1.2	-1.8 to +4.2	0.427	+0.2	-5.6 to +6.0	0.940	-1.1	-5.7 to +3.1	0.590	+1.3	-0.8 to +3.3	0.209
NN50 (number)	-22.5	-55.3 to + 10.3	0.168	**-86.2**	**-137.4 to -35.1**	**0.002**	**-57.5**	**-97.1 to -18.0**	**0.007**	+7.0	-17.1 to +31.2	0.550
PNN50 (%)	-83.0	-204.2 to + 38.1	0.168	**-318.2**	**-506.9 to -129.6**	**0.002**	**-212.4**	**-358.5 to -66.3**	**0.007**	+26.0	-63.2 to +115.2	0.550
Triangular index	+9.7	-4.7 to +24.1	0.176	+20.8	-5.9 to +47.6	0.120	-0.8	-21.7 to +20.2	0.941	-1.6	-12.3 to +9.1	0.758
TINN (ms)	+0.2	-0.2 to +0.7	0.245	+0.3	-0.6 to 1.2	0.493	+0.1	-0.7 to +0.7	0.996	+0.1	-0.2 to +0.5	0.366
**Frequency domain**												
Total power (ms^^2^^)	+0.1	-0.1 to 0.3	0.249	+0.1	-0.2 to +0.5	0.360	-0.1	-0.3 to +0.2	0.898	-0.1	-0.2 to +0.1	0.485
VLF (ms^^2^^)	+0.1	-0.1to +0.4	0.244	+0.3	-0.1 to +0.7	0.153	+0.1	-0.2 to +0.3	0.893	-0.1	-0.3 to +0.1	0.123
LF (nu)	+0.1	-0.4 to +0.5	0.772	+0.1	-0.7 to +1.0	0.727	+0.1	-0.5 to +0.7	0.803	-0.1	-0.4 to +0.2	0.553
HF (nu)	+0.6	-0.5 to + 0.4	0.799	-0.2	-1.1 to +0.7	0.637	-0.1	-0.8 to +0.5	0.669	+0.1	-0.2 to +0.4	0.509
LF/HF	+0.6	-12.9 to + 14.1	0.930	+4.5	-20.9 to 30.0	0.713	+2.2	-16.5 to 20.9	0.810	-3.7	-13.2 to +5.7	0.416
**Non-linear**												
SD1	+1.8	-2.5 to +6.0	0.388	-0.5	-8.7 to +7.7	0.898	-2.2	-8.2 to +3.6	0.439	+ 2.3	-0.6 to +5.1	0.116
SD2	+2.2	-0.3 to +4.8	0.085	+3.9	-1.0 to +8.9	0.116	+0.9	-2.9 to +4.8	0.612	+0.2	-1.7 to +2.2	0.800
SD1/SD2	+0.7	-41.9 to +43.3	0.973	-29.1	-108.4 to +20.2	0.453	-26.0	-83.9 to +31.9	0.359	+ 19.1	-9.7 to +47.9	0.182

CI=confidence interval; DBP=diastolic blood pressure; DP=double product; HF=high frequency component; HR=heart rate; HRV=heart rate variability; LF=low frequency component; LF/HF=low frequency/high frequency ratio (sympathovagal index); MAP=mean arterial pressure; NN50=number of consecutive normal RR interval differences > 50 ms; pNN50=percentage of normal RR intervals that differed by > 50 ms from the adjacent interval; RMSSD=root-mean-square of successive differences; SBP=systolic blood pressure; SD1=standard deviation of instantaneous beat-to-beat interval variability; SD2=continuous long-term RR interval variability; SD1/SD2=SD1/SD2 ratio; SDNN=standard deviation of normal RR interval; SF-36=36-item Short Form Health Survey; TINN=baseline width of the minimum square difference triangular interpolation of the highest peak of the histogram of all NN intervals; VLF=very low frequency component

**Table S2:** Associations between hemodynamics and heart rate variability variables with SF-36 Mental Composite domains (non-adjusted analysis).

	Vitality			Social functioning			Emotional role			Mental health		
	β	95% CI	*P*-value	β	95% CI	*P*-value	β	95% CI	*P*-value	β	95% CI	*P*-value
**Hemodynamics**												
HR (bpm)	+0.3	-0.8 to +1.5	0.536	-0.5	-1.3 to +0.3	0.215	-0.4	-1.3 to +0.4	0.281	-0.1	-1.0 to +0.7	0.723
**SBP (mmHg)**	-0.6	-1.31 to +0.1	0.075	-0.3	-0.8 to +0.2	0.283	-0.3	-0.8 to +0.2	0.220	-0.2	-0.7 to +0.3	0.345
DBP (mmHg)	-0.6	-1.7 to +0.6	0.306	-0.5	-1.3 to +0.3	0.186	-0.4	-1.3 to +0.4	0.279	+0.1	-0.8 to +0.9	0.877
MAP (mmHg)	-0.8	-1.8 to +0.3	0.138	-0.5	-1.2 to +0.2	0.184	-0.4	-1.2 to 0.3	0.210	-0.1	-0.9 to +0.6	0.721
**DP (bpm.mmHg)**	-0.1	-0.2 to 0.0	0.179	-0.1	-0.1 to +0.1	0.199	-0.1	-0.1 to +0.1	0.403	-0.1	-0.1 to +0.1	0.082
**HRV**												
**Time domain**												
Mean RR (ms)	-0.1	-0.2 to +0.1	0.202	+0.1	-0.1 to +0.1	0.625	-0.1	-0.1 to +0.1	0.660	-0.1	-0.1 to +0.1	0.534
SDNN (ms)	-0.6	-1.1 to +3.1	0.759	-0.1	-2.7 to +2.5	0.935	-0.4	-3.0 to +2.2	0.757	+1.0	-1.5 to +3.5	0.419
RMSSD (ms)	+1.0	-2.0 to +1.0	0.490	-0.1	-2.5 to +2.6	0.871	-1.7	-3.9 to +0.5	0.126	+ 1.5	-0.8 to +3.8	0.181
NN50 (number)	-12.7	-49.4 to +23.9	0.477	**-33.1**	**-54.8 to -11.5**	**0.005**	**-48.3**	**-62.2 to -34.4**	**<0.001**	-8.6	-34.8 to +17.6	0.502
PNN50 (%)	-47.0	-182.3 to +88.3	0.477	**-122.2**	**-202.1 to -42.4**	**0.005**	**-178.3**	**-229.6 to -127.1**	**<0.001**	-31.7	-128.5 to +65.0	0.502
Triangular index	-7.2	-23.1 to +8.7	0.357	+2.8	-8.8 to +14.4	0.620	+1.1	-10.4 to +12.7	0.840	+3.3	-8.2 to +14.8	0.553
TINN (ms)	+0.1	-0.4 to +0.7	0.647	+0.1	-0.4 to +0.4	0.859	-0.1	-0.5 to +0.3	0.584	+0.2	-0.2 to +0.6	0.387
**Frequency domain**												
Total power (ms^^2^^)	-0.1	-0.3 to +0.1	0.325	-0.1	-0.1 to +0.1	0.987	-0.1	-0.1 to +0.1	0.987	+0.1	-0.1 to +2	0.673
VLF (ms^^2^^)	-0.2	-0.4 to +0.1	0.112	+0.1	-0.1 to +0.2	0.755	+0.1	-0.1 to +0.2	0.334	-0.1	-0.2 to +0.2	0.872
LF (nu)	-0.3	-0.7 to +0.1	0.186	+0.1	-0.3 to +0.3	0.995	+0.1	-0.2 to +0.4	0.760	-0.1	-0.5 to +0.1	0.201
HF (nu)	+0.3	-0.2 to +0.8	0.235	-0.1	-0.3 to +0.3	0.876	-0.1	-0.4 to +0.3	0.695	+0.2	-0.1 to +0.5	0.256
LF/HF	-8.1	-22.1 to +6.0	0.247	+0.5	-9.9 to +11.0	0.917	+1.0	-9.4 to +11.4	0.845	-5.7	-15.7 to +4.4	0.252
**Non-linear**												
SD1	+0.9	-3.7 to +5.6	0.676	-0.4	-3.8 to +2.9	0.798	-2.7	-5.7 to +0.4	0.087	+1.8	-1.5 to +5.0	0.270
SD2	-0.6	-3.6 to +2.4	0.698	+0.4	-1.7 to +2.6	0.684	+0.4	-1.8 to +2.5	0.719	+0.9	-1.2 to +3.0	0.377
SD1/SD2	+18.1	-27.0 to +63.3	0.413	-6.1	-38.8 t +26.7	0.704	**-30.2**	**-59.8 to -0.7**	**0.045**	+11.7	-20.6 to +44.0	0.459

CI=confidence interval; DBP=diastolic blood pressure; DP=double product; HF=high frequency component; HR=heart rate; HRV=heart rate variability; LF=low frequency component; LF/HF=low frequency/high frequency ratio (sympathovagal index); MAP=mean arterial pressure; NN50=number of consecutive normal RR interval differences > 50 ms; pNN50=percentage of normal RR intervals that differed by > 50 ms from the adjacent interval; RMSSD=root-mean-square of successive differences; SBP=systolic blood pressure; SD1=standard deviation of instantaneous beat-to-beat interval variability; SD2=continuous long-term RR interval variability; SD1/SD2=SD1/SD2 ratio; SDNN=standard deviation of normal RR interval; SF-36=36-item Short Form Health Survey; TINN=baseline width of the minimum square difference triangular interpolation of the highest peak of the histogram of all NN intervals; VLF=very low frequency component

The associations of hemodynamics and HRV with QoL domains are depicted in Tables 2 and 3. SBP and DP were negatively associated with the physical functioning domain (Table 2). DP, NN50, and PNN50 exhibited negative association with the physical role domain (Table 2), while only NN50 and PNN50 were significantly associated with bodily pain (Table 2), social functioning, and emotional role domains (Table 3). For the vitality domain (Table 3), only SBP was negatively associated. Considering general (Table 2) and mental health (Table 3) domains, none of hemodynamic or HRV variables demonstrated significant association. Finally, DP, NN50, and PNN50 were negatively associated with the total score of QoL (Table 4).

**Table 2:** Associations of hemodynamics and heart rate variability variables with SF-36 Physical Composite domains.

	Physical Functioning			Physical Role			Bodily Pain			General Health		
	β	95% CI	*P*-value	β	95% CI	*P*-value	β	95% CI	*P*-value	β	95% CI	*P*-value
**Hemodynamics**												
HR (bpm)	-0.2	-1.6 to +1.2	0.758	-1.6	-4.2 to 1.0	0.201	-0.9	-3.0 to +1.1	0.355	+0.1	-0.1 to +1.0	0.954
SBP (mmHg)	**-0.6**	**-1.3 to 0.0**	**0.050**	-0.6	-2.0 to +0.7	0.343	-0.8	-1.8 to +0.2	0.126	-0.1	-0.6 to +0.4	0.724
DBP (mmHg)	-0.2	-1.4 to +0.9	0.681	-1.0	-3.2 to 13.9	0.326	-1.0	-2.6 to +0.7	0.234	-0.1	-0.8 to +0.8	0.947
MAP (mmHg)	-0.6	-1.6 to +0.4	0.248	-1.0	-2.9 to +0.9	0.293	-1.1	-2.5 to +0.4	0.145	-0.1	-0.8 to +0.7	0.835
DP (bpm.mmHg)	**-0.1**	**-0.1 to 0.0**	**0.045**	**-0.1**	**-0.1 to 0.0**	**0.006**	-0.1	-0.1 to +0.1	0.171	0.0	0.0 to +0.1	0.989
**HRV**												
*Time domain*												
Mean RR (ms)	+0.1	-0.2 to +0.1	0.315	+0.1	-0.2 to +0.4	0.671	-0.1	-0.3 to +0.1	0.461	-0.1	-0.1 to +0.1	0.428
SDNN (ms)	+1.1	-3.2 to +5.4	0.595	+1.0	-7.3 to +9.4	0.794	-0.4	-7.0 to +6.1	0.888	-0.2	-3.4 to +2.7	0.846
RMSSD (ms)	+0.3	-3.1 to +3.8	0.829	-1.0	-34.2 to +66.3	0.766	-1.5	-6.7 to +3.7	0.544	+0.9	-1.5 to +3.3	0.438
NN50 (number)	-29.4	-62.2 to +3.3	0.075	**-95.4**	**-145.4 to -45.4**	**0.001**	**-62.4**	**-106.8 to -18.0**	**0.009**	+1.3	-24.4 to +27.0	0.916
PNN50 (%)	-108.7	-229.6 to +12.2	0.075	**-352.0**	**-536.5 to -67.5**	**0.001**	**-230.3**	**-394.1 to -66.4**	**0.009**	+4.8	-90.0 to +99.7	0.916
Triangular index	+5.0	-12.0 to +22.0	0.545	+15.8	-16.6 to 48.2	0.318	-1.9	-28.0 to +24.2	0.882	-4.1	-16.2 to +8.0	0.484
TINN (ms)	+0.1	-0.5 to + 0.7	0.667	+0.2	-1.0 to +1.3	0.752	-0.1	-0.9 to +0.9	0.980	+0.3	-0.4 to +0.4	0.885
***Frequency domain***												
Total power (ms^^2^^)	+0.1	-0.2 to +0.2	0.697	0.0	-0.4 to +0.5	0.827	-0.1	-0.4 to +0.3	0.817	-0.1	-0.2 to +0.1	0.326
VLF (ms^^2^^)	+0.1	-0.2 to +0 .3	0.609	+0.2	-0.3 to 0.7	0.411	+0.1	-0.4 to +0.4	0.957	-0.1	-0.3 to +0.1	0.073
LF (nu)	-0.1	-0.6 to +0.3	0.684	-0.1	-1.0 to +0.8	0.795	+0.1	-0.6 to +0.8	0.867	-0.1	-0.5 to +0.2	0.444
HF (nu)	+0.1	-0.4 to +0.6	0.674	+0.1	-0.9 to 1.0	0.899	-0.1	-0.9 to +0.6	0.729	+0.1	-0.2 to +0.5	0.432
LF/HF	-3.0	-17.2 to +11.2	0.663	-1.6	-30.8 to 65.6	0.456	+1.7	-19.9 to + 23.4	0.866	-4.1	-14.1 to +5.9	0.397
***Nonlinear***												
SD1	+0.8	-3.8 to +5.4	0.707	-1.7	-10.6 t +7.2	0.692	-2.6	-9.5 to +4.3	0.438	+1.7	-1.5 to +4.9	0.281
SD2	+1.5	-2.3 to +5.2	0.419	+3.4	-3.9 to +10.7	0.335	+1.5	-4.3 to +7.3	0.585	-0.4	-3.1 to +2.3	0.765
SD1/SD2	+2.1	-41.8 to +46.1	0.919	-23.4	-107.7 to +60.9	0.566	-26.7	-92.0 to +38.5	0.399	15.9	-14.3 to +46.2	0.282

Statistical analysis was adjusted for age, gender, and time since surgery CI=confidence interval; DBP=diastolic blood pressure; DP=double product; HF=high frequency component; HR=heart rate; HRV=heart rate variability; LF=low frequency component; LF/HF=low frequency/high frequency ratio (sympathovagal index); MAP=mean arterial pressure; NN50=number of consecutive normal RR interval differences > 50 ms; PNN50=percentage of normal RR intervals that differed by > 50 ms from the adjacent interval; RMSSD=root-mean-square of successive differences; SBP=systolic blood pressure; SD1=standard deviation of instantaneous beat-to-beat interval variability; SD2=continuous long-term RR interval variability; SDNN=standard deviation of normal RR interval; SF-36=36-item Short Form Health Survey; TINN=baseline width of the minimum square difference triangular interpolation of the highest peak of the histogram of all NN intervals; VLF=very low frequency component

**Table 3:** Associations of hemodynamics and heart rate variability variables with SF-36 Mental Composite domains.

	Vitality			Social Functioning			Emotional Role			Mental Health		
	β	95% CI	*P*-value	β	95% CI	*P*-value	β	95% CI	*P*-value	β	95% CI	*P*-value
**Hemodynamics**												
HR (bpm)	+0.3	-1.3 to +1.9	0.684	-0.7	-1.7 to +0.4	0.207	-0.3	-1.4 to +0.7	0.501	-0.1	-1.3 to +1.0	0.793
SBP (mmHg)	**-0.7**	**-1.5 to +0.1**	**0.047**	-0.2	-0.7 to +0.4	0.504	-0.3	-0.8 to +0.3	0.328	-0.3	-0.9 to +0.3	0.354
DBP (mmHg)	-0.7	-1.9 to +0.6	0.280	-0.5	-1.4 to +0.4	0.243	-0.4	-1.3 to +0.4	0.305	+0.1	-0.9 to +1.0	0.907
MAP (mmHg)	-0.9	-2.0 to +0.2	0.110	-0.4	-1.2 to +0.4	0.304	-0.4	-1.2 to +0.4	0.275	-0.1	-1.0 to +0.7	0.715
DP (bpm.mmHg)	-0.1	-0.1 to +0.1	0.277	-0.1	-0.1 to +0.1	0.172	-0.1	-0.1 to +0.1	0.210	-0.1	-0.1 to +0.1	0.849
**HRV**												
*Time domain*												
Mean RR (ms)	-0.1	-0.2 to +0.1	0.153	+0.1	-0.1 to +0.1	0.797	-0.1	-0.1 to +0.1	0.651	-0.1	-0.2 to +0.1	0.560
SDNN (ms)	-0.6	+5.5 to +4.3	0.787	-1.0	-4.4 to +2.4	0.525	-1.1	-4.4 to +2.2	0.495	+1.6	-1.9 to +5.1	0.343
RMSSD (ms)	+0.8	-2.7 to +4.4	0.632	-0.4	-3.2 to +2.3	0.736	-2.0	-4.5 to 0.5	0.104	+1.9	-0.9 to +4.6	0.168
NN50 (number)	-18.4	-58.5 to + 21.7	0.346	**-36.0**	**-58.0 to -14.0**	**0.003**	**-48.2**	**-62.0 to -34.4**	**<0.001**	-8.4	-38.4 to +21.6	0.562
PNN50 (%)	-68.0	-215.9 to +80.0	0.346	**-132.7**	**-213.9 to -51.5**	**0.003**	**-178.0**	**-228.8 to -27.1**	**<0.001**	-31.0	-141.6 to +79.6	0.562
Triangular index	-8.1	-27.3 to +11.2	0.388	+0.6	-13.1 to +14.3	0.923	-0.4	-13.9 to +13.1	0.956	+4.3	-10.0 to +18.6	0.537
TINN (ms)	+0.1	-0.6 to +0.8	0.781	-0.1	-0.5 to +0.5	0.933	-0.1	-0.5 to +0.4	0.731	+0.2	-0.2 to +0.7	0.300
*Frequency domain*												
Total power (ms^^2^^)	-0.1	-0.4 to +0.1	0.410	-0.1	-0.2 to +0.1	0.589	-0.1	-0.2 to +0.1	0.567	+0.1	-0.1 to +0.2	0.686
VLF (ms^^2^^)	-0.2	-0.5 to +0.1	0.167	-0.1	-0.2 to +0.2	0.861	+0.1	-0.1 to +0.2	0.581	-0.1	-0.2 to +0.2	0.837
LF (nu)	-0.3	-0.8 to +0.2	0.258	-0.1	-0.5 to +0.3	0.592	-0.1	-0.4 to +0.4	0.972	-0.2	-0.6 to +0.1	0.203
HF (nu)	+0.3	-0.3 to +0.8	0.332	+0.1	-0.3 to +0.5	0.725	-0.1	-0.4 to +0.4	0.987	+0.2	-0.2 to +0.6	0.252
LF/HF	-7.2	-23.1 to +8.7	0.354	-1.6	-13.0 to +9.7	0.764	-0.8	-12.1 to +10.3	0.868	-6.4	-18.0 to +5.1	0.257
*Nonlinear*												
SD1	+0.6	-4.6 to +5.9	0.791	-0.7	-4.4 to +2.9	0.680	-2.7	-9.6 to +0.7	0.110	+2.1	-1.6 to +5.9	0.251
SD2	-0.7	-5.1 to +3.7	0.725	-0.1	-3.2 to +3.0	0.940	+0.1	-2.9 to +3.1	0.929	+1.7	-1.4 to +4.8	0.268
SD1/SD2	+14.1	-35.6 to +63.8	0.557	-4.7	-39.5 to +30.2	0.781	-26.3	-58.1 to +5.3	0.097	+13.5	-22.7 to +49.8	0.441

Statistical analysis was adjusted for age, gender, and time since surgery CI=confidence interval; DBP=diastolic blood pressure; DP=double product; HF=high frequency component; HR=heart rate; HRV=heart rate variability; LF=low frequency component; LF/HF=low frequency/high frequency ratio (sympathovagal index); MAP=mean arterial pressure; NN50=number of consecutive normal RR interval differences > 50 ms; PNN50=percentage of normal RR intervals that differed by > 50 ms from the adjacent interval; RMSSD=root-mean-square of successive differences; SBP=systolic blood pressure; SD1=standard deviation of instantaneous beat-to-beat interval variability; SD2=continuous long-term RR interval variability; SDNN=standard deviation of normal RR interval; SF-36=36-item Short Form Health Survey; TINN=baseline width of the minimum square difference triangular interpolation of the highest peak of the histogram of all NN intervals; VLF=very low frequency component

**Table 4:** Associations of hemodynamics and heart rate variability variables with SF-36 total score.

Variable	β	95% CI	*P*-value
**Hemodynamics**			
HR (bpm)	-0.4	-1.6 to +0.7	0.434
SBP (mmHg)	-0.4	-1.0 to + 0.1	0.118
DBP (mmHg)	-0.5	-1.4 to +0.5	0.307
MAP (mmHg)	-0.6	-1.4 to +0.3	0.172
DP (bpm.mmHg)	**-0.1**	**-0.1 to -0.0**	**0.039**
**HRV**			
***Time domain***			
Mean RR (ms)	-0.1	-0.2 to +0.1	0.569
SDNN (ms)	0.1	-3.7 to +3.7	0.985
RMSSD (ms)	-.126	-3.1 to +2.9	0.929
NN50 (number)	**-37.1**	**-61.6 to-12.6**	**0.005**
PNN50 (%)	**-137.0**	**-227.3 to -46.7**	**0.005**
Triangular index	+1.4	-13.4 to +16.2	0.843
TINN (ms)	+0.1	-0.4 to +0.6	0.777
*Frequency domain*			
Total Power (ms^^2^^)	-0.1	-0.2 to +0.2	0.805
VLF (ms^^2^^)	-0.1	-0.2 to +0.2	0.946
LF (nu)	-0.1	-0.5 to +0.3	0.563
HF (nu)	+0.1	-0.3 to +0.5	0.662
LF/HF	-2.9	-15.1 to +9.3	0.624
*Nonlinear*			
SD1	-0.3	-4.3 to +3.7	0.874
SD2	+0.9	-2.4 to +4.2	0.581
SD1/SD2	-4.4	-42.1 to +33.3	0.807

Statistical analysis was adjusted for age, gender, and time since surgery CI=confidence interval; DBP=diastolic blood pressure; DP=double product; HF=high frequency component; HR=heart rate; HRV=heart rate variability; LF=low frequency component; LF/HF=low frequency/high frequency ratio (sympathovagal index); MAP=mean arterial pressure; NN50=number of consecutive normal RR interval differences > 50 ms; PNN50=percentage of normal RR intervals that differed by > 50 ms from the adjacent interval; RMSSD=root-mean-square of successive differences; SBP=systolic blood pressure; SD1=standard deviation of instantaneous beat-to-beat interval variability; SD2=continuous long-term RR interval variability; SDNN=standard deviation of normal RR interval; SF-36=36-item Short Form Health Survey; TINN=baseline width of the minimum square difference triangular interpolation of the highest peak of the histogram of all NN intervals; VLF=very low frequency component

## DISCUSSION

The main finding of the present study was the negative association of hemodynamic and autonomic function indexes with QoL in HT recipients. The benefits of heart transplantation for health-related QoL are well described for several domains/areas such as physical, psychological, and social^[14^-^16]^ but the role of hemodynamic and autonomic function variables as predictors of QoL has never been previously described. Briefly, the main variables exhibiting its association were SBP, DP, NN50, and PNN50.

Considering hemodynamics, the higher the SBP, the worse the physical function and vitality domains of QoL. Both elevated BP and total peripheral resistance and also attenuated BP and total peripheral resistance responses during orthostatic maneuvers are observed in HT recipients, probably due low-pressure cardiopulmonary baroreceptor denervation. Moreover, efferent sympathetic denervation leads to diminished cardiac output response to isometric exercise after heart transplantation. Together, those physiological impairments impose negative functional consequences to HT recipients^[17]^ that could explain the observed negative association of SBP with domains of QoL. In addition, as observed in the present study, the higher the DP, the worse the physical function and physical role domains of QoL. As an index of myocardial oxygen consumption previously used during exercise testing in patients with coronary heart disease, DP reflects cardiac workload, being a well-established index of energy consumption of heart^[18]^. Therefore, increases in DP at rest could indicate a small DP reserve (difference between rest and maximal exercise DP) that has greater prognostic power than metabolic equivalents, maximal HR, or SBP^[19]^, reflecting the efficiency of the myocardium^[20]^. Then, elevated resting DP could impair physical function - and by consequence, the clinical status of those patients - due to elevated resting myocardial oxygen consumption and low exercise capacity by reduced cardiac reserve^[21]^, worsening QoL.

Considering autonomic function, NN50 and PNN50 (indexes associated with parasympathetic activity^[22]^, more reliable in short-term observations^[23]^) increases were contradictorily associated with reduction of physical role, bodily pain, social functioning, and emotional role domains and total score. PNN50 was already correlated to decreases in physical function^[24]^.

Despite the expected improvement in QoL due to increased parasympathetic activation, that could ameliorate hemodynamics by reducing resting HR, SBP, and finally DP, improving DP reserve and, as consequence, cardiac reserve and exercise capacity, which would result in clinical improvements, it was not demonstrated in the present study. Since parasympathetic fibers only innervate nodal peacemakers’ cells, the main influence of its activation should be the stimulation of HR reduction, or more precisely, the inhibition of HR elevation due to humoral adrenergic stimulation^[25^,^26]^.

In fact, parasympathetic influence on HR is absent in the majority of patients up to eight years after cardiac transplantation^[27]^, as are in the population of the present study, in which mean time since transplantation was near four years. Furthermore, HR exhibited no association with QoL domains. A possible explanation to such contradiction could be the peripheral effect of parasympathetic nerve stimulation by releasing the neurotransmitter acetylcholine on muscular and endothelial layers of blood vessels, that induces smooth muscle cells contraction, leading to increase of peripheral resistance that, at last, increases SBP^[28]^. It can be demonstrated by the impairment of cardiac performance during exercise in orthotopic HT recipients, in which acute beta-adrenergic blockade accentuates the impairment in ventricular performance and appears to be detrimental in these patients, probably by reduction in myocardial contractility and increase in peripheral resistance^[7]^. Thus, this pathophysiological mechanism evidences the impact of ANS imbalance (previously indicating both parasympathetic and sympathetic activation^[29]^) on physical and emotional functions, which can worse social interactions. Finally, the elevated adrenergic stimulation and plasma circulating catecholamine levels, already associated to pain syndromes via adrenergic receptors stimulation, are suggested in HT recipients^[29^,^30]^, being a possible mechanism underlining the inverse association of parasympathetic activation and bodily pain QoL domain^[31]^.

Beyond physiological, laboratorial, or clinical variables and morbidities, mortality, and other classical medical hard outcomes, QoL - which reflects “the individual’s perception of their insertion in life, in the context of the culture and value systems in which a person lives and in relation to their goals, expectations, standards and concerns” - is a major outcome for patients’ life, so the majority of healthcare assistance treatment strategy should be focused on its improvement, ensuring that the treatment is centered on the patient rather than the disease^[32^,^33]^.

However, in clinical practice, it is not easy to assess QoL, mainly because its subjectivity, but also the complexity of health-related QoL questionaries, and the difficulty for some patients in understanding and answering it. So, based on the results, the main implication for practice was the identification of some hemodynamic and autonomic variables associated with QoL, which may provide a less subjective way for corroborate the interpretation of this relevant outcome, and finally improve clinical management. Despite the need for more research about those associations, maybe the clinical approach of those variables should impact in improvement in QoL for HT patients. So, as noninvasive, cheap, and easy to perform, HRV - a promising tool for clinically monitoring of autonomic dysfunction prior to several cardiovascular disease occurrence and also for monitoring cardiovascular disease progression^[34^,^35]^ - together with hemodynamic basic measurements, such as BP and DP, could be more than an instrument for ambulatorial clinical follow-up, perhaps being added as clinical therapeutic targets for improving QoL (the main objective of heart transplantation), as suggested by our results.

### Limitations

We consider a study limitation the small sample size and the lack of follow-up of those patients to understand temporal evolution of this association. We are also aware that this is a single-center study, and our results may not apply to other settings.

## CONCLUSION

The present study demonstrated DP and HRV as predictors of QoL in HT recipients. These innovative results can become a relevant therapeutic target for improving QoL in HT recipients prior to its deterioration.

**Table:** 

Authors’ Roles & Responsibilities	
LFRJ	Substantial contributions to the conception or design of the work; and the acquisition, analysis, and interpretation of data for the work; drafting the work and revising it critically for important intellectual content; agreement to be accountable for all aspects of the work in ensuring that questions related to the accuracy or integrity of any part of the work are appropriately investigated and resolved; final approval of the version to be published
BRM	Substantial contributions to the conception or design of the work; and the acquisition, analysis, and interpretation of data for the work; agreement to be accountable for all aspects of the work in ensuring that questions related to the accuracy or integrity of any part of the work are appropriately investigated and resolved; final approval of the version to be published
APD	Drafting the work and revising it critically for important intellectual content; agreement to be accountable for all aspects of the work in ensuring that questions related to the accuracy or integrity of any part of the work are appropriately investigated and resolved; final approval of the version to be published
JRO	Substantial contributions to the conception or design of the work; and the acquisition, analysis, and interpretation of data for the work; agreement to be accountable for all aspects of the work in ensuring that questions related to the accuracy or integrity of any part of the work are appropriately investigated and resolved; final approval of the version to be published
PHSF	Drafting the work and revising it critically for important intellectual content; agreement to be accountable for all aspects of the work in ensuring that questions related to the accuracy or integrity of any part of the work are appropriately investigated and resolved; final approval of the version to be published
CRO	Drafting the work and revising it critically for important intellectual content; agreement to be accountable for all aspects of the work in ensuring that questions related to the accuracy or integrity of any part of the work are appropriately investigated and resolved; final approval of the version to be published
ASC	Drafting the work and revising it critically for important intellectual content; agreement to be accountable for all aspects of the work in ensuring that questions related to the accuracy or integrity of any part of the work are appropriately investigated and resolved; final approval of the version to be published
MFFM	Substantial contributions to the conception or design of the work; and the acquisition, analysis, and interpretation of data for the work; drafting the work and revising it critically for important intellectual content; agreement to be accountable for all aspects of the work in ensuring that questions related to the accuracy or integrity of any part of the work are appropriately investigated and resolved; final approval of the version to be published
TCFG	Substantial contributions to the conception or design of the work; and the acquisition, analysis, and interpretation of data for the work; drafting the work and revising it critically for important intellectual content; agreement to be accountable for all aspects of the work in ensuring that questions related to the accuracy or integrity of any part of the work are appropriately investigated and resolved; final approval of the version to be published

## References

[1] Albuquerque DC, Neto JD, Bacal F, Rohde LE, Bernardez-Pereira S, Berwanger O (2015). I Brazilian registry of heart failure - clinical aspects, care
quality and hospitalization outcomes. Arq Bras Cardiol.

[2] Agnetti G, Piepoli MF, Siniscalchi G, Nicolini F. (2015). New insights in the diagnosis and treatment of heart
failure. Biomed Res Int.

[3] Emin A, Rogers CA, Banner NR, Steering Group, UK Cardiothoracic Transplant Audit (2016). Quality of life of advanced chronic heart failure: medical care,
mechanical circulatory support and transplantation. Eur J Cardiothorac Surg.

[4] Nicolini F, Piepoli MF, Agnetti G, Siniscalchi G. (2015). Alternatives to transplantation in the treatment of heart
failure: new diagnostic and therapeutic insights. Biomed Res Int.

[5] Harris C, Cao C, Croce B, Munkholm-Larsen S. (2018). Heart transplantation. Ann Cardiothorac Surg.

[6] Bacal F, Neto JD, Fiorelli AI, Mejia J, Marcondes-Braga FG, Mangini S (2010). II Diretriz Brasileira de Transplante Cardíaco [II
Brazilian Guidelines for Cardiac Transplantation]. Arq Bras Cardiol.

[7] Verani MS, Nishimura S, Mahmarian JJ, Hays JT, Young JB. (1994). Cardiac function after orthotopic heart transplantation: response
to postural changes, exercise, and beta-adrenergic blockade. J Heart Lung Transplant.

[8] Grupper A, Gewirtz H, Kushwaha S. (2018). Reinnervation post-heart transplantation. Eur Heart J.

[9] Moreira BR, Duque AP, Massolar CS, de Lima Pimentel R, Mediano MFF, Guimarães TCF (2019). Transcutaneous electrical stimulation of PC5 and PC6 acupoints
modulates autonomic balance in heart transplant patients: a pilot
study. J Acupunct Meridian Stud.

[10] Campolina AG, Bortoluzzo AB, Ferraz MB, Ciconelli RM. (2011). Validação da versão brasileira do
questionário genérico de qualidade de vida short-form 6
dimensions (SF-6D Brasil). Cien Saude Colet.

[11] Pekmezović T, Ječmenica-Lukić M, Petrović I, Špica V, Tomić A, Kostić VS. (2015). Quality of life in patients with progressive supranuclear palsy:
one-year follow-up. J Neurol.

[12] Kim HG, Cheon EJ, Bai DS, Lee YH, Koo BH. (2018). Stress and heart rate variability: a meta-analysis and review of
the literature. Psychiatry Investig.

[13] Roy B, Ghatak S. (2013). Nonlinear methods to assess changes in heart rate variability in
type 2 diabetic patients. Arq Bras Cardiol.

[14] Grady KL. (2003). Quality of life after heart transplantation: are things really
better?. Curr Opin Cardiol.

[15] Grady KL, Jalowiec A, White-Williams C. (1998). Quality of life 6 months after heart transplantation compared
with indicators of illness severity before transplantation. Am J Crit Care.

[16] Trevisan ME, Porto AS, Pinheiro TM. (2010). Influência do treinamento da musculatura
respiratória e de membros inferiores no desempenho funcional de
indivíduos com DPOC. Fisioter Pesq.

[17] Nygaard S, Christensen AH, Rolid K, Nytrøen K, Gullestad L, Fiane A (2019). Autonomic cardiovascular control changes in recent heart
transplant recipients lead to physiological limitations in response to
orthostatic challenge and isometric exercise. Eur J Appl Physiol.

[18] Nelson RR, Gobel FL, Jorgensen CR, Wang K, Wang Y, Taylor HL. (1974). Hemodynamic predictors of myocardial oxygen consumption during
static and dynamic exercise. Circulation.

[19] Sadrzadeh Rafie AH, Sungar GW, Dewey FE, Hadley D, Myers J, Froelicher VF. (2008). Prognostic value of double product reserve. Eur J Cardiovasc Prev Rehabil.

[20] Domka-Jopek E, Jopek A, Bejer A, Lenart-Domka E, Walawski G. (2018). The importance of the double product in the six-minute walk test
to predict myocardial function. Biomed Res Int.

[21] Sniecinski RM, Skubas NJ, London MJ. (2012). Testing cardiac reserve: then and now. 1923. Anesth Analg.

[22] Mietus JE, Peng CK, Henry I, Goldsmith RL, Goldberger AL. (2002). The pNNx files: re-examining a widely used heart rate variability
measure. Heart.

[23] Shaffer F, Ginsberg JP. (2017). An overview of heart rate variability metrics and
norms. Front Public Health.

[24] Hathaway DK, Wicks MN, Cashion A, Milstead EJ, Cowan PA, Gaber AO. (1998). Posttransplant improvement in heart rate variability correlates
with improved quality of life. West J Nurs Res.

[25] Hasan W. (2013). Autonomic cardiac innervation: development and adult
plasticity. Organogenesis.

[26] Gordan R, Gwathmey JK, Xie LH. (2015). Autonomic and endocrine control of cardiovascular
function. World J Cardiol.

[27] Arrowood JA, Minisi AJ, Goudreau E, Davis AB, King AL. (1997). Absence of parasympathetic control of heart rate after human
orthotopic cardiac transplantation. Circulation.

[28] Sheng Y, Zhu L. (2018). The crosstalk between autonomic nervous system and blood
vessels. Int J Physiol Pathophysiol Pharmacol.

[29] Yoshitatsu M, Ohtake S, Sawa Y, Fukushima N, Nishimura M, Sakakida S (2000). Assessment of autonomic reinnervation of cardiac grafts by
analysis of heart rate variability. Transplant Proc.

[30] Guimarães GV, D'Avila V, Bocchi EA, Carvalho VO. (2010). Norepinephrine remains increased in the six-minute walking test
after heart transplantation. Clinics (Sao Paulo).

[31] Carroll I, Mackey S, Gaeta R. (2007). The role of adrenergic receptors and pain: the good, the bad, and
the unknown. Semin Anesth.

[32] The World Health Organization (1995). Quality of Life assessment (WHOQOL): position paper from the
World Health Organization. Soc Sci Med.

[33] Higginson IJ, Carr AJ. (2001). Measuring quality of life: using quality of life measures in the
clinical setting. BMJ.

[34] Sessa F, Anna V, Messina G, Cibelli G, Monda V, Marsala G (2018). Heart rate variability as predictive factor for sudden cardiac
death. Aging (Albany NY).

[35] Kubota Y, Chen LY, Whitsel EA, Folsom AR. (2017). Heart rate variability and lifetime risk of cardiovascular
disease: the atherosclerosis risk in communities study. Ann Epidemiol.

